# Evaluation of the accuracy of conventional and digital implant impression techniques in bilateral distal extension cases: a randomized clinical trial

**DOI:** 10.1186/s12903-024-04495-0

**Published:** 2024-07-05

**Authors:** Wafaa Youssef Elashry, Mohamed Maamoun Elsheikh, Ali Mohamed Elsheikh

**Affiliations:** 1 Assistant lecturer, Prosthodontic department, Faculty of Dentistry, Tanta University, Tanta, Egypt; 2 Professor, Prosthodontic department, Faculty of Dentistry, Tanta University, Tanta, Egypt

**Keywords:** Implant impression, Accuracy of impression, Digital workflow, Intraoral scanning

## Abstract

**Background:**

This clinical study aims to evaluate the accuracy of the conventional implant impression techniques compared to the digital impression ones in bilateral distal extension cases.

**Methods:**

A total of 32 implants were placed in eight patients missing all mandibular posterior teeth except the first premolars. Each patient received a total of four implants, with two implants placed on each side, in order to provide support for three units of screw-retained zirconia restorations. Following osteointegration, the same patient underwent two implant-level impression techniques: Conventional open-tray impressions CII (splinted pick-up) and digital implant impressions DII with TRIOS 3 Shape intraoral scanner. The accuracy of impressions was evaluated utilizing a three-dimensional superimposition analysis of standard tessellation language (STL) files. Subsequently, the scan bodies were segmented using Gom inspect software to measure three-dimensional deviations in a color-coding map. Data were statistically analyzed using the Kruskal Wallis test and then a post-hoc test to determine the significance level (*P* < 0.05).

**Results:**

The study revealed that higher angular and positional deviations were shown toward distal scan bodies compared to mesial ones for both impression techniques. However, this difference was not statistically significant (*P* > 0.05).

**Conclusion:**

Splinted open-tray conventional impression and intraoral scanning implant impression techniques have demonstrated comparable accuracy.

**Trial registration:**

Clinical Trials.gov Registration ID NCT05912725. *Registered 22/06/ 2023- Retrospectively registered*, https://register.clinicaltrials.gov.

**Supplementary Information:**

The online version contains supplementary material available at 10.1186/s12903-024-04495-0.

## Background

Oral rehabilitation utilizing implant-supported prosthesis for patients with partial dentition has been recognized as a highly effective therapeutic option [1]. The clinical passivity of the implant-supported prosthesis and the accuracy of the prosthodontic workflow are critical determinants of the long-term efficacy of this treatment modality [2, 3].

The implant impression is considered one of the crucial steps in fabricating a well-fitting implant prosthesis. Therefore, making accurate implant impressions is mandatory because the inaccurate transfer of 3D implant locations leads to the ill-fitting prosthesis with biological (peri-implantitis) and mechanical complications such as screw loosening, screw fracture, prosthesis or implant fracture, and other adverse effects [4, 5].

Various impression techniques have been used to fabricate accurate implant-supported prosthesis. The Conventional impression is an usual technique for recording the actual position of the implant from the patient’s mouth to the master cast for a long time. However, multiple variables influence its accuracy, including impression material and technique type, tray selection, impression coping design, number of implants, angulation, implant connection type, cast pouring, and dental stone expansion [6, 7].

The conventional workflow entails several sequential actions that elevate the likelihood of human error at any phase. Furthermore, these errors are unavoidable and will compromise the precision of the final prosthesis supported by dental implant [8].

The aforementioned factor incited researchers to seek a viable alternative technique to conventional workflow. The advent and growing popularity of digital technology have enabled clinicians to employ intraoral scanners and intra-oral scan bodies (ISBs) to overcome the obstacles and inconveniences associated with conventional impression materials and techniques [9, 10].

The digital impression generates a digital scan from the patient’s mouth directly. A digital implant analog can be inserted into a virtual digital model utilizing implant/ISB libraries once the image has been captured precisely [11]. Therefore, direct digitalization has advantages over the conventional method, such as improving patient satisfaction due to the elimination of impression material and tray selection [12].

Furthermore, it is more comfortable and regarded as the optimal choice for patients with severe gag reflexes, breathing difficulties, or allergies to impression materials [13, 14]. Additionally, information can be electronically shared and stored as digital data. Any missing data or artifacts can also be rescanned, which significantly reduces total working time. In contrast, the conventional technique would require a new impression [15, 16].

Nevertheless, several factors can hinder the performance and accuracy of the IOS. These include scan body design, the position of the scanning area, scanning strategy, the operator’s experience, soft wares calibrations, excessive salivation, blood presence, size and movement of the tongue, mobile tissue, humidity, room temperature and light condition as ambient lighting is a factor of interest which has been reported to influence intraoral scanning accuracy [17]. The digital scans are becoming more and more popular for partially edentulous jaw and are also emrging for complete edentulous patients. Most studies conduced to assess the accuracy of impression techniques were in vitro under controlled conditions, and systematic reviews comparing the digital impressions with conventional impressions in terms of accuracy in fixed restorations concluded that short, fixed dental prostheses from digital impressions could be clinically acceptable and DII has been deemed a viable alternative compared to conventional procedures. As all data in the literature are numerous and controversial, there is an urgent need for valid clinical data on conventional and digital implant impressions [18, 19].

Therefore, this clinical study aimed to evaluate the accuracy of conventional implant impressions compared to digital impressions. Consequently, we can test the null hypothesis that there is no significant difference in accuracy between the two methods for patients with free-end distal extension.

## Methods

Following the approval of the Research Ethics Committee (RP-9-20-1), the study’s objective was explained to the patients and informed consent was signed before the conduction of the study. In addition, informed consent was obtained according to the guidelines on human research adopted by the Research Ethics Committee, Faculty of Dentistry, Tanta University, and the study was registered on www.clinicaltrial.gov (22/06/2023 - ID: *NCT05912725*).

### Eligibility criteria

The inclusion criteria in the study were adequate bone length and width to accommodate the implant, sufficient interarch space, and overall good health of the patients. The patient’s general health was assessed by taking a complete medical history to exclude any systemic conditions that might have impaired implant osseointegration.

### Patient allocation

A randomized clinical trial was conducted on eight patients with bilateral missing all mandibular posterior teeth (mandibular molars and second premolar) except for the first premolars, which were the last remaining teeth. The study’s subjects were selected from patients attending the outpatient clinic of the Prosthodontics Department at Faculty of Dentistry, Tanta University. The sample size calculated using a computer program G power version 3.1.9. Cone beam CTs were carried out for all participants to check the quality and quantity of bone and to detect any abnormality for Implant planning and construction of the surgical guides.

**Fig. 1:**
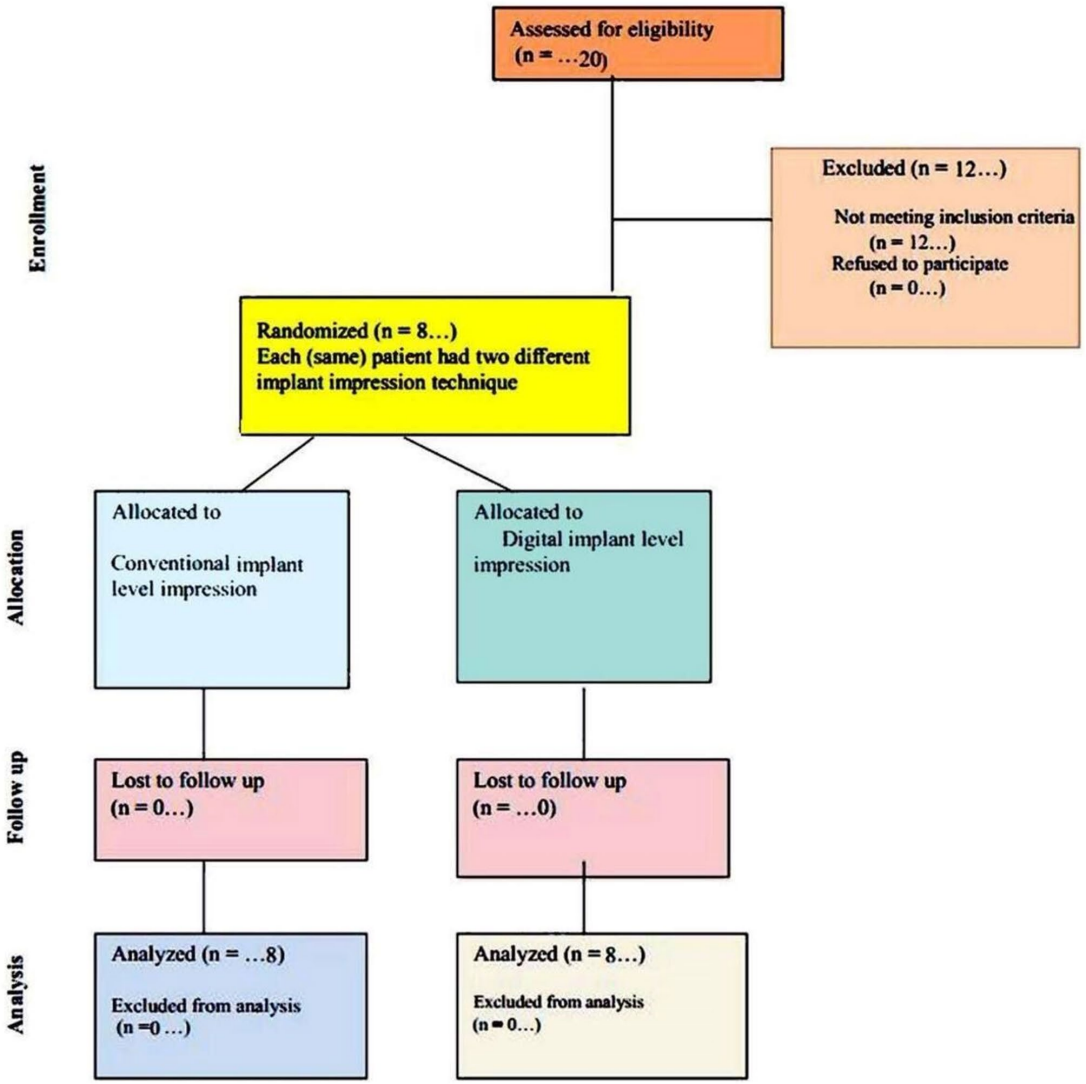
CONSORT flow diagram

Four dental implants (CopaSKY, Bredent Medical GmbH& Co.KG) were inserted in each patient through a surgical guide. Two implants were on each side opposite the second premolar and second molar, to support three units of screw-retained zirconia restorations. The same qualified implantologist did all the surgical procedures. 3 months after acquisition of osteointegration, the same patient underwent two distinct implant-level impression techniques (*n* = 8) for each technique (Fig. 1).

### Digital implant level impression (DII)

**Fig. 2:**
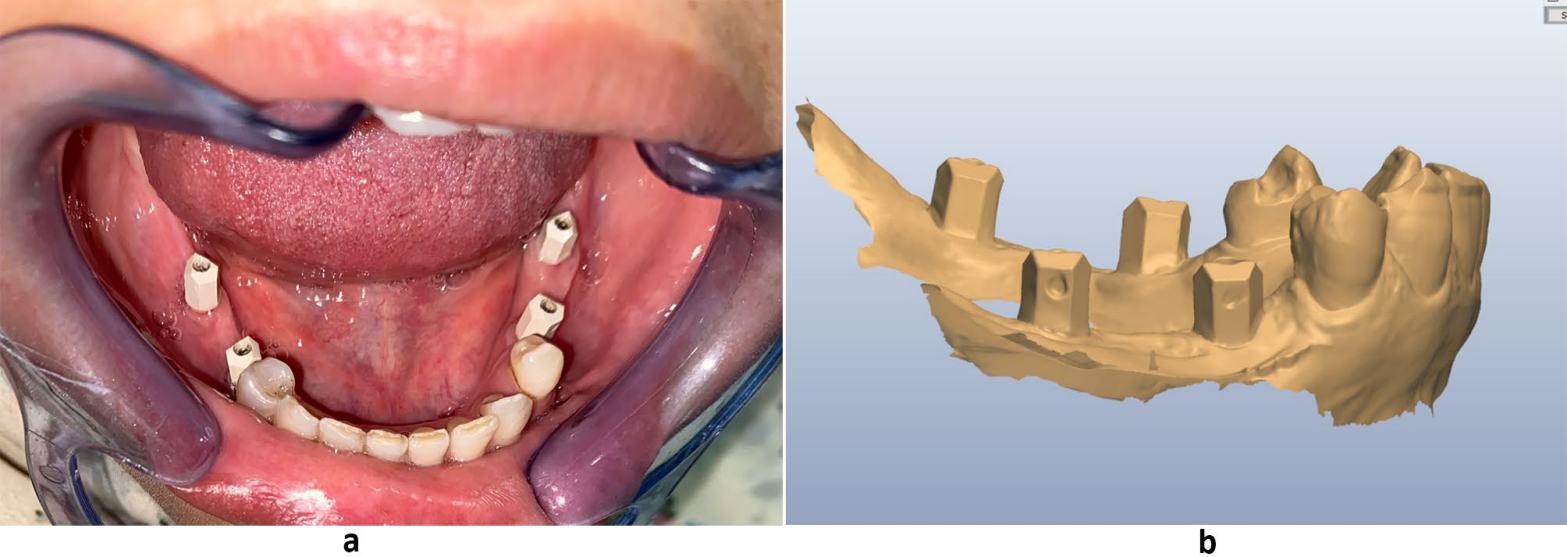
**(a)** Digital implant impression. Peek scan bodies. **(b)** Digital model showing notches on the vertical surface of scan bodies

A digital impression was captured utilizing Trios 3Shape IOS (version 1.3.4.2) after securely attaching the peek scan bodies (Bredent Medical, GmbH& Co.KG) to the fixtures at 15 Ncm. For standardization, notch marks were prepared on the vertical surface of the scan body provided that were directed toward the buccal side during intraoral scanning to ensure that the same scan bodies have the same orientation and position on the cast during extraoral scaning to facilitate the superimposition of both STL files of conventional and digital impressions (Fig. 2a, b).

The scanning technique was performed continuously, starting from the posterior scan bodies on the patient’s right side and moving in a zigzag motion through the anterior teeth, concluding at the posterior scan bodies on the patient’s left side. Following the Trios 3 shape IOS protocol, this scanning strategy involved scanning the occlusal surface first, then the lingual surface, and finally the buccal surface. The digital image underwent a thorough examination, and any gaps in the data were promptly filled by rapidly scanning using a scanner [20].

### Conventional implant level impressions (CII)

**Fig. 3:**
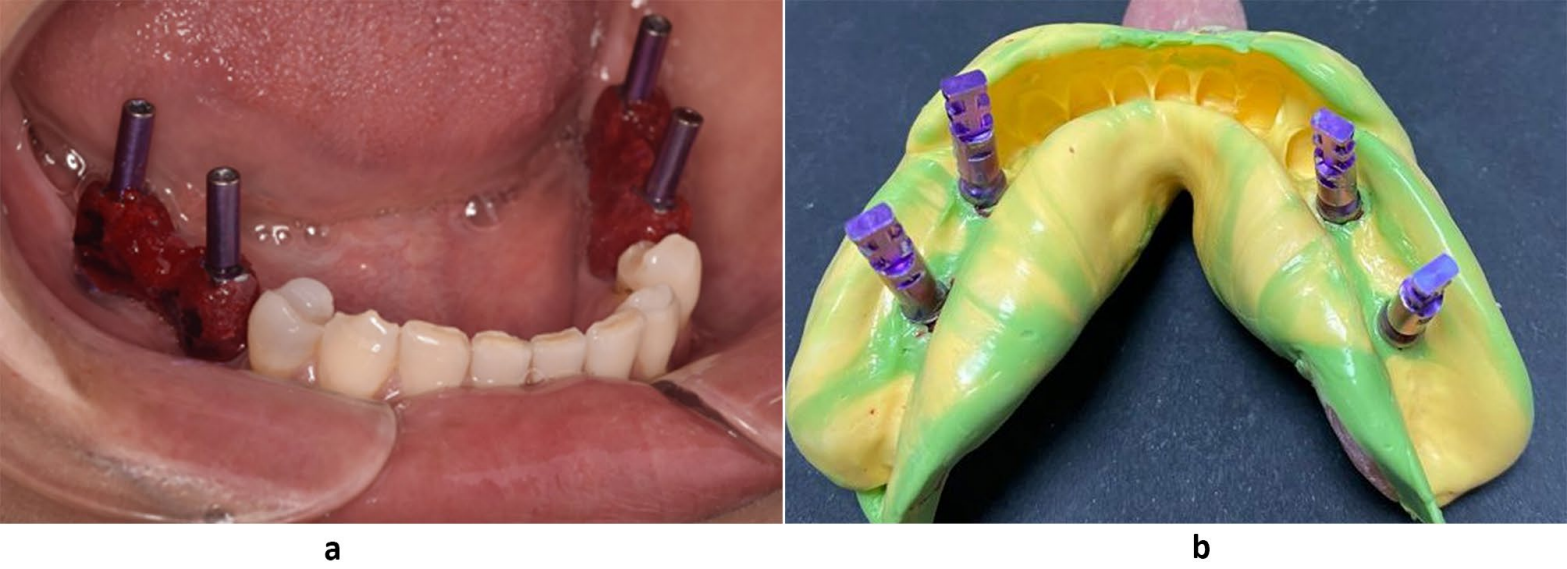
**(a)** Splinting of pick-up copings. **(b)** Polyvinyl siloxane conventional impression

Following the removal of the healing abutments, open tray impression copings (Bredent Medical, GmbH& Co.KG) were affixed to the fixtures. Periapical X-rays were taken to ensure that the copings were securely positioned, then the primary impression was taken with properly adjusted stock trays then implant analogues were attached to impression copings and casts were poured with type IV stone. Impression copings were attached to the fixture analogues embedded in the cast. A splinting procedure of impression copings and their verification Jig were performed with orthodontic wire and duralay resin on the cast in the dental laboratory .The splinted assemblies were sectioned with carborundum disc through the middle of the index in the dental lab and Custom-made acrylic impression trays were constructed with 4 windows corresponding to each implant site, before final impression taking, the pick-up impression copings were secured to the implants and periapical x-ray was performed for every side to confirm that each impression post was completely seated on its own position within fixture and their proper fit was confirmed using a passivity verification Jig, then reconnected and rejoined again with freshly mixed resin material by the brush bead method intraorally to minimize polymerization shrinkage of this resin material as recommended by Cerqueira et al. [21] (Fig. 3a).

The custom -made open tray was coated with thin layer of adhesive then impressions were made using poly vinyl-siloxane (PVS) impression material, specifically Zhermack Elite HD + S.p. A, following the manufacturer’s guidelines. After the impression material was set, all copings were unscrewed and separated from the implants. In addition, the impression tray was removed vertically from the mouth, following the long axis to minimize lateral stresses [22].

The implant analogs (Bredent Medical, GmbH& Co.KG) were securely tightened on top of the copings embedded with the impression material (Fig. 3b), The silicone ginigival mask material (Bredent, GmbH& Co.KG) was injected around four implant analogs.The master casts were poured with type IV dental stone (SHERAPREMIUM super hard stone, type 4, gold braun GmbH&Co.KG.) and allowed to set at room temperature for 24 h before digitization. Then the conventional stone casts were scanned with an extraoral scanner (DOF, Freedom HDSeongdong-gu) using the same peek scan bodies at the same locations and orientation as what happened with the digital impression .

### The digital evaluation of accuracy between conventional and digital implant-level impression techniques

**Fig. 4:**
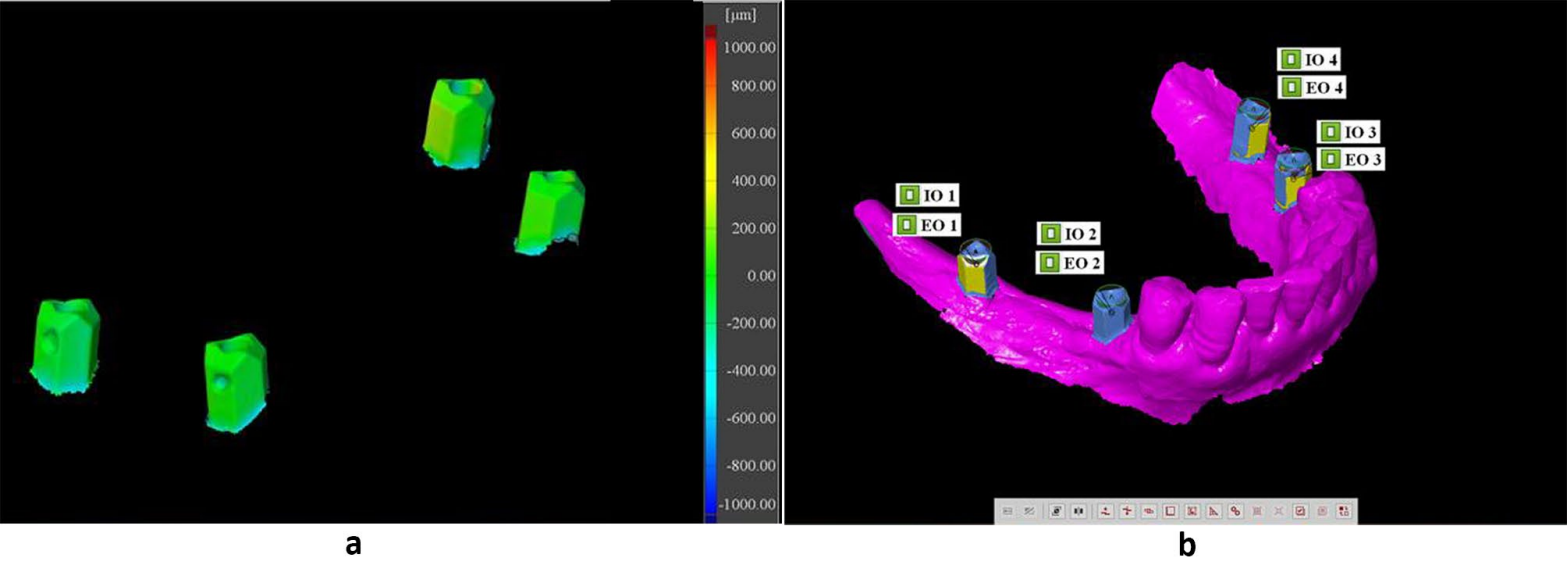
**(a)** Segmentation method of scan bodies. **(b)** Fitting element inside each scan body

Prior to importing into the 3D analysis program software (GOM Inspect 2016, Gom GmbH), all STL files underwent examination to identify any intersections and eliminate redundant data. This study supposed and served the digital implant impression as the reference data for subsequent comparisons. A conventional impression technique was used as a test method, with the aid of anterior natural teeth and the incisal edge serving as reference points. This facilitated the alignment of conventional STL files onto STL files obtained from digital scans. Once the best-fit alignment was achieved, the scan body part was segmented to measure the three-dimensional angular deviations of scan bodies in degrees between the two techniques. Additionally, the 3D positional deviations were recorded in micrometers (µm) and represented using a color coding map (green, blue, and red). As demonstrated in (Fig. 4a), the green color indicated that the surfaces were identical [23, 24]. The distance between the two scan bodies’ centers was measured in millimeters as the inter-implant distance.

Subsequently, the scan bodies are labeled sequentially, beginning with the most posterior scan body (opposite the second molar) on the patient’s right side, denoted as letter **A**. The anterior scan body (second premolar) on the right side is named **B**, while on the left, the anterior scan body represents the second premolar taking **C**, and the posterior scan body taking **D** looks like a **u** shape.

Four cylinders were created using fitting elements within each scan body. This allowed for the creation of four cylinders for intraoral scanning and four cylinders for extraoral scanning bodies (Fig. 4b). Each cylinder was then compared and assessed against its corresponding counterpart, denoted by the same letter. For instance, the cylinder from extraoral scan body **A** was compared with the corresponding cylinder from intraoral scan body **A**, and so forth. The central axis of each scan body was determined by identifying the point of intersection between the longitudinal axis and the horizontal plane, which was created on the upper horizontal surface of the scan body according to Flügge et al. [25].

### Measurement

Subsequently, by the blinded examiner the three-dimensional deviations were recorded among superimposed scan bodies between both impression techniques. The angular deviation of scan bodies in the vertical axis was measured in (degree) as the angle formed when the implant deviated from its long axis.

For the evaluation of positional deviation of scan bodies was measured in (µm), the vertical edge of the scan body was used as how much the platform of implant moved in x axis, y axis, and z axis and overall.

**Fig. 5:**
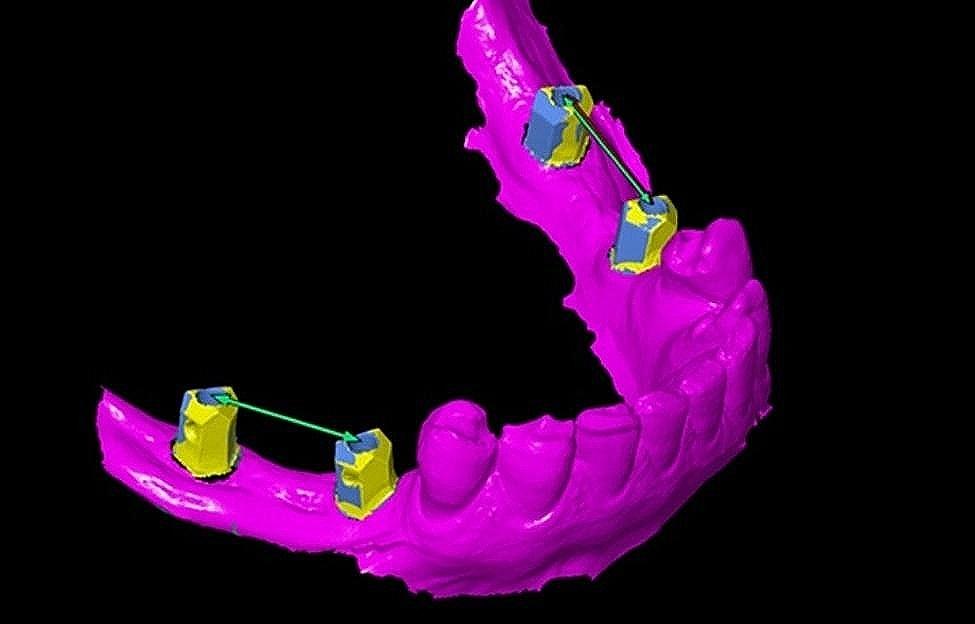
Inter-implant distance between central points of two scan bodies

The distance between central points of neighboring scan bodies was recorded in the same side of each technique and compared with their counterpart of the other impression technique in millimeters (Fig. 5).

### Statistical analysis

Data were collected and analyzed statistically using (IBM SPSS software, version 20.0.) to the significance level of 0.05.

## Results

The Shapiro-Wilk test of normality showed that not all data were distributed normally. Therefore, the Kruskal-Wallis test was utilized to compare the angular and positional deviations of superimposed scan bodies **(A, B, C, D)** between conventional and digital implant impression techniques.

Table (1) lists the mean and standard deviation of angular deviation of scan bodies between CII and DII impression techniques: for scan body **A** was (2.34 ± 1.71°) while (1.17 ± 0.59°) for scan body **B** and (2.24 ± 1.06°) for scan body **C**. The highest mean angular deviation value was observed in scan body **D** (2.94 ± 1.78°). There were no significant differences (*P* = 0.095).

**Fig. 6:**
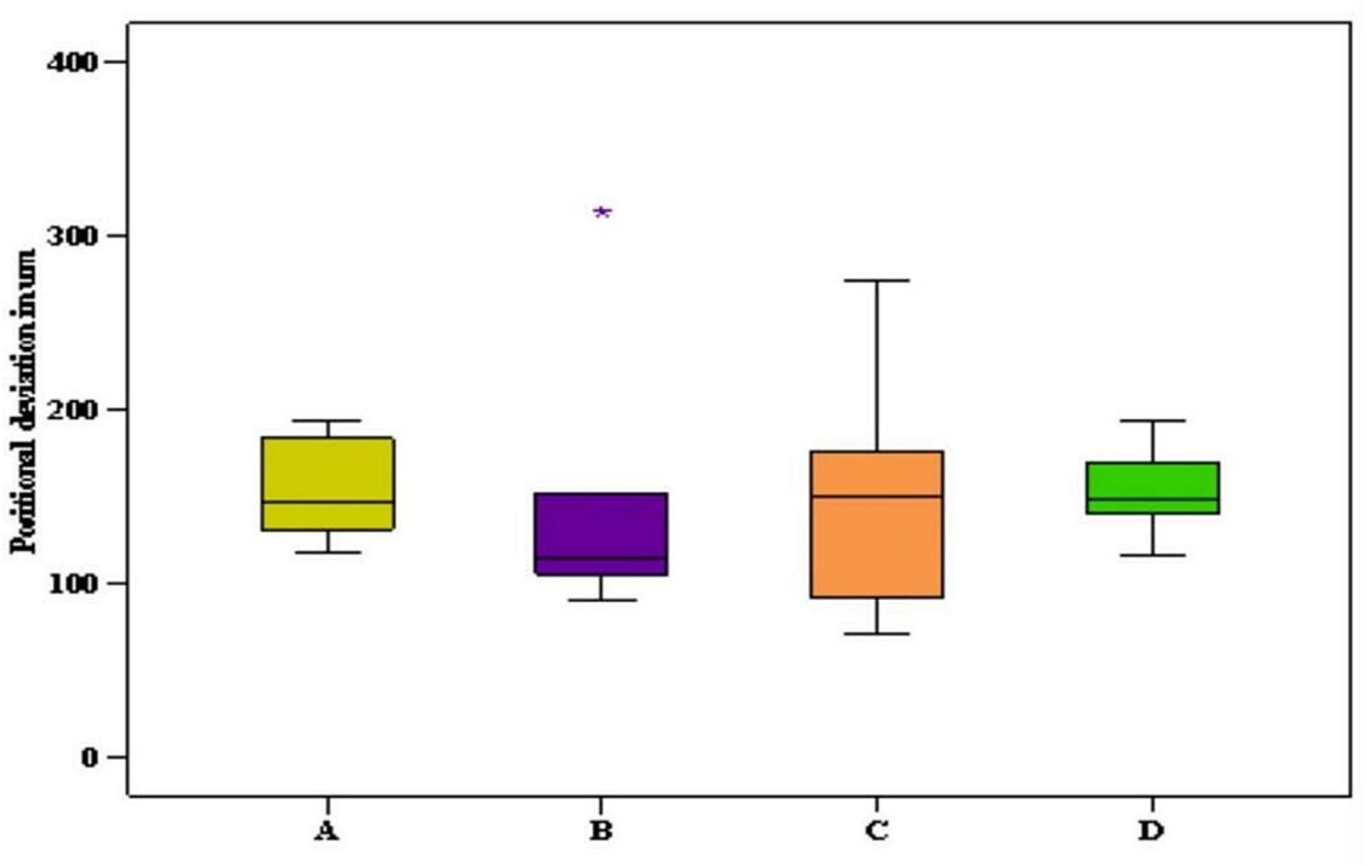
Positional deviation between conventional and digital impressions in µm

The positional deviation of scan bodies in the x, y, and z axes between conventional and digital implant impressions was analyzed. The scan bodies with the highest positional deviations were **A** (154.04 ± 29.98 μm) and **D** (153.14 ± 25.49 μm). Scan body **B** had a deviation of 143.15 ± 72.41 μm, while scan body **C** had a deviation of 147.49 ± 66.35 μm. These deviations were deemed clinically acceptable and did not exhibit a statistically significant difference, with *p* > 0.05 (Fig. 6).

The comparison between distal (posterior) scan bodies and mesial (anterior) scan bodies revealed that distal scan bodies exhibited a greater angular deviation (2.64 ± 1.71°) compared to mesial ones (1.71 ± 0.99°), as illustrated in Table 1.

**Table 1:** Mean of angular deviation of scan bodies between conventional and digital impressions

	The angular deviation between both impressions in degree				H	*p*	Mean Angular deviation of posterior scan bodies versus anterior ones		U	*p*
	A	B	C	D			posterior (A–D) (*n* = 16)	Interior (B–C) (*n* = 16)		
**Min.**	0.21	0.29	0.89	1.00	6.368	0.095	0.21	0.29	0.86	0.119
**Max.**	4.86	2.32	3.54	5.96			5.96	3.54		
**Mean**	2.34	1.17	2.24	2.94			2.64	1.71		
**SD.**	1.71	0.59	1.06	1.78			1.71	2.55		

**U**: Mann Whitney test. **SD**: Standard deviation. **H**: H for Kruskal Wallis test

**p:** *p* value for comparing between the studied groups

Regarding inter-implant distance measurements between the centers of two scan bodies, the results of the Student t-test showed no significant difference between conventional and digital impression *P* > 0,05 in Table 2.

**Table 2:** Mean of inter-implant distance between conventional and digital impressions in (mm)

	Conventional impression		Digital impression	
	A–B	C–D	A–B	C–D
**Min.**	12.61	12.62	12.61	12.63
**Max.**	22.43	18.10	21.79	17.52
**Mean**	17.02	15.20	16.91	15.18
**SD.**	3.73	2.13	3.74	1.95
**P**			0.927	0.961

**SD:** Standard deviation

**P:***P*-value for Paired t-test for comparing between Conventional impression and Digital impression in each patient

## Discussion

The key finding of this study indicated that digital implant impression is an effective alternative to the conventional method in manufacturing 3-unit fixed partial dentures. Both techniques exhibit comparable levels of accuracy and fall within the range considered clinically acceptable, which is consistent with prior studies [26, 27].

The digital impression was utilized as the reference data in this clinical study, as it has proven to overcome challenges associated with analog impression techniques and streamline both clinical and laboratory procedures. This finding is in agreement with Ting-Shu S and Jian S [28] and Ahlberg et al. [29]. They concluded that digital workflow was more accurate than conventional workflow. In contrast, Alsharbaty et al. [30] illustrated that the conventional pick-up impression is the most accurate technique.

The TRIOS 3 shape IOS software was calibrated, and notches were made on the vertical surface of scan bodies provided that directed buccally during scanning for standardization.

The results of the present study showed there were angular and positional deviations among superimposed scan bodies between conventional and digital implant impression techniques with no statistically significant difference. When comparing distal scan bodies with mesial scan bodies, it was noticed that they had higher mean angular deviation (2.64 ± 1.71 ^o^) than mesial ones(1.71 ± 0.99 ^o^). This finding can be attributed to the difficulty during impression-taking in the posterior region due to poor accessibility. Nevertheless, these deviations fell within the range of clinically acceptable values and did not exceed the acceptable threshold, according to Jemt. The acceptable range is stated to be up to 150 μm, and another study found that the trueness should be less than 200 μm [24, 31].

The observed differences were significantly lower when the standard deviation was high; this could potentially be ascribed to clinical variation, as Alsharbaty et al. [30]. Consequently, a high standard deviation of the detected differences should be considered. Maximum values could have clinical significance.

A one-step polyvinyl siloxane (PVS) material was used as it demonstrated accuracy in minimizing distortion. A systematic review conducted by Lee et al. [7] showed no significant differences between PVS and polyether. Conversely, Parameshwari et al. [32] suggested that Polyvinyl Siloxane impression material is more accurate than polyether impressions in a partially edentulous arch.

The GOM inspect software was used to import all STL files for superimposition in order to calculate the total 3D deviation. This deviation was represented using a color coding map. During the superimposition process, the color green indicates that both techniques are identical. It has been observed that the accuracy of the outcome is significantly influenced by the superimposition and measurement methods. Therefore, the segmentation method plays a crucial role in overcoming this problem [13].

Best fit alignment offers the advantage of minimizing the average error, which is a measure of the accuracy of surface alignment. Additionally, it decreases the overall distances between the scan bodies in the test and their corresponding counterparts in the reference data found in the implant library [11].

The limitations of this study include that no reliable and standered clinical reference data.

## Conclusions

In this clinical study, it was found that there was no significant difference in accuracy between conventional and digital implant impressions for cases where implants were used to support partially edentulous patients. Additionally, both techniques showed 3D deviations that were within clinically acceptable levels. However, different study designs are recommended to valid reference data for clinical trials.

### Clinical relevance

Intraoral digital implant impression using scan body displays better performance and time saving and materials than conventional implant impression techniques for recording the actual position of posterior implant in bilateral distal extension cases.

### Electronic Supplementary Material

Below is the link to the electronic supplementary material.

Supplementary Material 1

Supplementary Material 2

Supplementary Material 3

## Data Availability

The datasets used and/or analyzed during the current study are available from the corresponding author upon reasonable request.

## Declarations

### Supplementary Information

The full trial protocol is available and can be accessed in the related file section and consort 2010 check list is uploaded on the system .
