# Supplementary material for: Evaluation of the accuracy of conventional and digital implant impression techniques in bilateral distal extension cases: a randomized clinical trial
Source: BMC Oral Health. 2024 Jul 5;24:764. doi: 10.1186/s12903-024-04495-0 (PMC11227137; doi:10.1186/s12903-024-04495-0)
Supplement: Supplementary file 1 — Supplementary Material 1 [file 12903_2024_4495_MOESM1_ESM.docx]

- **Title**: Evaluation of the accuracy of conventional and digital implant impression techniques in bilateral distal extension cases: A randomized clinical trial

The approval of the Research Ethics Committee NO (RP-9-20-1)

- **The investigators:**

**Wafaa Youssef Elashry:** Assistant lecturer, Prosthodontic department, Faculty of Dentistry, Tanta University, Tanta, Egypt.

**Mohamed Maamoun Elsheikh**: Professor, Prosthodontic department, Faculty of Dentistry, Tanta University, Tanta, Egypt.

**Ali Mohamed Elsheikh:** Professor, Prosthodontic department, Faculty of Dentistry, Tanta University, Tanta, Egypt.

- **Research site:** Faculty of Dentistry, Tanta University, Tanta, Egypt.

Address: Tanta, El-Gaish St. Medical Campus - Faculty of Dent

Phone: 0403318537

FAX: 040/ 3452319 – Ext. 160

Postal Code: 31773

E-MAIL: [dean_dent@unv.tanta.edu.eg](mailto:dean_dent@unv.tanta.edu.eg)

- **Funding: Self-funded research**
- **Abstract:**
- **Background:** This clinical study aims to evaluate the accuracy of the conventional implant impression techniques compared to the digital impression ones in bilateral distal extension cases**.**
- **Methods:** A total of 32 implants were placed in eight patients missing all mandibular posterior teeth except the first premolars. Each patient received a total of four implants, with two implants placed on each side, in order to provide support for three units of screw-retained zirconia restorations. Following osteointegration, the same patient underwent two implant-level impression techniques: Conventional open-tray impressions CII (splinted pick-up) and digital implant impressions DII with TRIOS 3 Shape intraoral scanner. The accuracy of impressions was evaluated utilizing a three-dimensional superimposition analysis of standard tessellation language (STL) files. Subsequently, the scan bodies were segmented using Gom inspect software to measure three-dimensional deviations in a color-coding map. Data were statistically analyzed using the Kruskal Wallis test and then a post-hoc test to determine the significance level (*P* <0.05).
- **Results:** The study revealed that higher angular and positional deviations were shown toward distal scan bodies compared to mesial ones for both impression techniques. However, this difference was not statistically significant (*P* >0.05).
- **Conclusion**: Splinted open-tray conventional impression and intraoral scanning implant impression techniques have demonstrated comparable accuracy.
- **Trial registration:** *Clinical Trials.gov Registration: ID: NCT05912725.*
- *Registered 22/06/ 2023- Retrospectively registered*, [*https://register.clinicaltrials.gov*](https://register.clinicaltrials.gov)

**Background information:**

Today, the use of dental implants to rehabilitate partially and completely edentulous patients is a routine procedure. Numerous clinical studies have demonstrated the long-term efficacy of this modality. Despite the prognosis is expected to be good with a success rate of approximately 97-99%, there is still a chance of failures. They are mostly attributed to the inaccurate surgical or prosthodontic technique.^(1)^

Implant supported restorations offer considerable advantages when compared to removable partial dentures, improved support, a more secure occlusion, preservation of bone and simplicity of the prosthesis are a few reasons why implants are the treatment of choice for missing posterior teeth. The clinical passivity of implant-supported prosthesis is affected by several factors during the construction procedure, including the implant impression technique, master cast fabrication, wax pattern creation, framework fabrication, definitive prosthesis construction, and definitive prosthesis delivery, since each step in the manufacturing process is directly influenced by the previous phase.^(2)^

Accuracy of the implant impression is a crucial factor to achieve highly successful implant supported prosthesis with passive fit which in turn affects the final outcome. Basic impressions techniques for implant prosthesis can be either the implant level or the abutment level. Traditional implant level impressions are a universal technique and can be classiﬁed into two categories: direct or indirect techniques. ^(3)^ Direct techniques are also known as open tray impression because the tray has an open window for unscrewing the guide pins of the impression copings, the whole set is removed at the same time, and the copings are repositioned by ﬁxation of this same screw. While indirect techniques are also known as closed tray impression procedures. These techniques utilize transfers that stay on the implants even after the impression tray is removed from the mouth. The transfer is unscrewed from the implant, and tightened to the analog outside the mouth, and placed back onto the impression, and may differ from case to case, as the closed tray technique is indicated in such cases: difﬁculty in accessibility of posterior implants, nausea and limited inter arch space. ^(4)^

The most commonly used technique to transfer the implant position from the intraoral cavity to the dental cast is the conventional impression techniques; however, all current traditional techniques result in some degree of error manifested as displacement of the implant analogs in the definitive cast compared with the digital intraoral positioning of the implants.

More recently, with the advent of computer-aided design and computer aided manufacturing (CAD/CAM) technology, it is now possible to use a digital workﬂow when fabricating implant-supported restorations which can be either direct or indirect in nature. The indirect workﬂow needs taking a traditional implant impression then digitized by using laboratory scan bodies and an optical bench top scanner. While the direct workﬂow includes the use of intraoral scan body (ISBs) and an intraoral scanning device to generate a digital impression directly from the patient’s mouth. ^(5)^

Digital implant impressions (DIIs) using the intraoral scanners (IOSs) became a convenient alternative for the analogue impression technique .The application of intraoral scanners (IOSs) in the field of fixed and implant prosthodontics offer several advantages over the conventional pathway, such as the elimination of tray selection; impression material, reduced risk of distortion during impression making, pouring, disinfecting, shipping to the dental laboratory and improving patient satisfaction.^(6,7)^

In addition to improving efﬁciency of the digital workflow and decreasing costs, digital scans can be transferred and stored electronically as digital information for the manufacturing of definitive prosthetic restorations The digital scan will produce a virtual cast that may be converted into a physical cast via milling or printing if a combination of digital and conventional workﬂow is required. Despite the rapid development of digital implant impression as an alternative to the conventional one, on technique has yet been recognized as the gold standard.

**The rationale of this study**:

Despite there are multiple invitro studies and few in vivo studies have been carried out to compare the conventional impression and intraoral scanning, there is no standard implant impression technique and no reference data in clinical scenario, so our research team supposed that the digital impression with a TRIOS 3 shape using scan bodies has been considered as a reference data as a control group due to its reality by recording actual position of the dental implant directly from the patient mouth avoiding the impression material with its drawbacks, while the conventional impression as a test group .

**The Objective of this study:** To evaluate the accuracy of conventional implant impressions (CII) and digital implant impressions (DII) in patients with bilateral distal extension cases and indicate which one is better.

**Study Design**: This study will be conducted as a crossover randomized clinical trial.

**Ethical Considerations**: Prior to the commencement of the study, ethical approval will be obtained from the Research Ethics Committee of Faculty of Dentistry, Tanta University NO (RP-9-20-1), the purpose of the current study was explained to the patients and Informed consent will be obtained from all participants before their inclusion in the study.

**Clinical study**:

- Inclusion Criteria:
  - - Patients’ age ranging from 30 to 55 years old.
    - Patients with bilateral missing all mandibular posterior teeth (mandibular molars and second premolar) except for the first premolars.
    - Adequate bone height and width to fulfill the criteria for implant placement.
    - They have acceptable maxillomandibular relationship and sufficient inter arch space.
    - The opposing arch was almost dentulous and any missing teeth were restored using fixed partial denture.
- Exclusion Criteria:
  - - Patients with systemic conditions that might impair soft or hard tissue healing.
    - Parafunctional habits.
    - Heavy smoking.

**Methodology**

**Sample Size Calculation**: The sample size will be determined using a computer program G Power version 3.1.9 based on previous studies and the desired level of statistical power.

**Preliminary radiographic examination**

Panoramic radiographs were carried out for all patients to exclude any pathology, bone abnormality, impacted teeth, or remaining roots in the area of implant sites and to ensure presence of adequate bone above the inferior alveolar canal.

**Cone beam computed tomography (CBCT)^[[1]](#footnote-1)^ was done for all the selected cases for:**

1. Accurate estimation of bone quality and quantity at implant sites.
2. Implant planning.
3. Construction of the surgical guide stent.

**Participants**:

Eight patients who had missing all mandibular posterior teeth bilaterally except first premolars as last standing teeth were included in this study. In each patient, 4 implant were placed ,2 in each side to support 3 units screw retained zirconia restorations. Three months after osteointegration each patient underwent two different types of implant level impression techniques (n=8): Conventional implant impressions (CII) using open tray with splinted copings, and digital implant impressions (DII) were taken with TRIOS 3Shape intraoral optical scanner (IOS), as each patient has two STL files of impressions, one from conventional impression and the other one from digital impression.

**Interventions**:

- Conventional Implant Impressions (CII):
  - Open tray impression copings will be affixed to the implants.
  - Polyvinyl siloxane (PVS) impression material will be used for making the impressions.
- Digital Implant Impressions (DII):
  - Trios 3Shape IOS will be utilized for capturing digital impressions.
  - Peek scan bodies will be securely attached to the fixtures at the implant level.

***Digital evaluation of accuracy of conventional implant impression technique versus digital impression technique:***

The digital impression with a TRIOS 3 shape using scan bodies considered as a reference while the test group was the open tray conventional impression with splinted copings.

1- STL files were captured by the IOS and the laboratory scanner were imported into a three-dimensional analysis program^[[2]](#footnote-2)^* GOM Inspect software to assess the accuracy of conventional impression versus digital implant impression by using superimposition and calculate the three-dimensional deviations of scan bodies between intraoral scanning (reference) and conventional impression (test) that were illustrated in color coding map in µm.

The green color meant perfectly matching surfaces; the red meant the conventional impression was positively positioned relative to reference digital impression while, the blue color meant test STL file was negatively positioned relative to reference STL file.

2- To ensure a precise superimposition, each imported STL file was checked for the presence of any redundant (un necessary), crossing and unstable faces in the imported data.

3- For surface superimposition first initial alignment, second best fit algorithm then 3D compares of the models carried out into 2 parts: (reference data and measure data) anterior natural teeth and incisal edge were used as a reference point facilitating surface superimposition between the models.

4- Then segmentation of scan bodies separately and removing useless data for 3D comparison of accuracy between the two impression techniques, and calculate 3 D deviation in micrometer (µm) in y-, x-, and z-directions.

5- After that, naming of the scan bodies starting from most posterior scan body in right side taking letter **A**, and anterior scan body in right side **B**, while on the left side anterior scan body representing second premolar and posterior scan body taking **C** and **D** respectively looks like u shape.

6-Four cylinders were constructed using fitting elements inside each scan body to obtain 4 cylinders for intraoral and 4 cylinders for extraoral scan bodies, through these fitting elements each cylinder was compared and evaluated with itself which having the same number for example: Cylinder of extra oral scan body **no. A** compared with such one of intraoral scan body **no. A** and so on, Subsequently, the deviations between the measured distances in the scan datasets and the reference aid were calculated.

7- For final alignment, a best fit algorithm was performed minimizing the arithmetic mean error. Therefore, it was possible to visualize three-dimensional deviations via surface comparison on CAD data and to examine the deviations of the spheres in y-, x-, and z-directions.

8-To identify the scan body axis, the section plane was created on the scan body shell. This section plane is perpendicular to the cylinder part direction and a reference vector normal to this reference plane is the axis of the scan body. The center point of the scan body is at the intersection between this axis and the top plane.

9- The angular deviation of scan bodies in vertical axis were recorded by measuring the angle between the circles and cylinders at each implant site, in test scans and the reference scan and also can expressed as the implant itself deviated from its long axis.

10- Positional deviation was recorded as how much the platform of implant moved x axis, y axis, and z axis and overall.

11- For linear measurement: the inter-implant distance between center points of two scan bodies in the same side described as the length of the straight line that connects these two points was measured for each impression technique and compared with each other.

**References:**

1. Papaspyridakos P,Gallucci GO, Chen CJ, Hanssen S, Naert I, Vandenberghe B. Digital versus conventional implant impressions for edentulous patients: accuracy outcomes. Clin Implant Dent Relat Res 2016;27(4):465-72.
2. Wee AG, Aquilino SA, Schneider RL. Strategies to achieve fit in implant prosthodontics: a review of the literature. Int J Prosthodont. 1999;12(2).
3. Alsharbaty MHM, Alikhasi M, Zarrati S, Shamshiri AR. A clinical comparative study of 3‐dimensional accuracy between digital and conventional implant impression techniques. J Prosthodont 2019;28(4):902-8.
4. Rudolph H, Salmen H, Moldan M, Kuhn K, Sichwardt V, Wöstmann B, et al. Accuracy of intraoral and extraoral digital data acquisition for dental restorations. J Appl Oral Sci. 2016;24:85-94.
5. Sharma S, Agarwal S, Sharma D, Kumar S, Glodha N. Impression; Digital vs. conventional: A review. Ann Dent Spec. 2014;2(1):9-10.
6. Flügge T, van der Meer WJ, Gonzalez BG, Vach K, Wismeijer D, Wang P. The accuracy of different dental impression techniques for implant‐supported dental prostheses: A systematic review and meta‐analysis. Clin Oral Implants Res. 2018;29:374-92.
7. Dohiem MM, Abdelaziz MS, Abdalla MF, Fawzy AM. Digital assessment of the accuracy of implant impression techniques in free end saddle partially edentulous patients. A controlled clinical trial. BMC Oral Health 2022;22(1):486.

**Outcome Measures**:

- Primary Outcome: Angular and positional deviations of superimposed scan bodies (A, B, C, D) between conventional and digital implant impression techniques.
- Secondary Outcome: Inter-implant distance measurements between the centers of two scan bodies.

**Data Collection**:

- Three-dimensional deviations will be recorded among superimposed scan bodies using GOM Inspect 2016 software.
- Angular deviation of scan bodies in the vertical axis will be measured in degrees.
- Positional deviation of scan bodies in x, y, and z axes will be measured in micrometers.
- Inter-implant distance measurements will be recorded in millimeters.

**Statistical Analysis**: Data will be collected and analyzed using IBM SPSS software, version 20.0, with a significance level set at 0.05.

**Expected Results**: We anticipate that both conventional and digital implant impressions will demonstrate comparable accuracy in patients with bilateral distal extension cases.

**Conclusion**: This study aims to provide valuable insights into the accuracy of conventional and digital implant impressions, which will contribute to optimizing the prosthodontic workflow in implant dentistry.

**Clinical relevance:** Intraoral digital implant impression using scan body displays better performance and time saving and materials than conventional implant impression techniques for recording the actual position of posterior implant in bilateral distal extension cases

1. Scanora 3D, Soredex Co., Tuusula, Finland. [↑](#footnote-ref-1)
2. * GOM Inspect 2016, Gom GmbH, Braunschweig, Germany. [↑](#footnote-ref-2)
