# Supplementary material for: Evaluation of the accuracy of conventional and digital implant impression techniques in bilateral distal extension cases: a randomized clinical trial
Source: BMC Oral Health. 2024 Jul 5;24:764. doi: 10.1186/s12903-024-04495-0 (PMC11227137; doi:10.1186/s12903-024-04495-0)
Supplement: Supplementary file 3 — Supplementary Material 3 [file 12903_2024_4495_MOESM3_ESM.pdf]

Tanta University
Faculty of Dentistry
Research Ethics Committee

Informed Consent Form for Participation in Research

I acknowledge that:

I have read a detailed statement about the research submitted by doctor/

Department of at the Faculty of Dentistry, Tanta University

I voluntarily agree to participate in this research, which aims to study ……………

and will be conducted during period from ………… to …………. I agree to the division of the groups mentioned in the research and the possibility of my random distribution to these groups, and I was informed of any new experience in this research.

I agree to the research steps and the resulting benefits, which are:

I have been informed about the potential difficulties that may arise from the study, which are

I have been also informed about the alternatives that will be followed in the event of these risks, as follows"

Other additions:

My refusal to continue participating in the research at any later time will not result in the withholding of any medical services provided to me, provided that the results of this study are kept strictly confidential and not used for any purpose other than scientific research.

I have read the above information or it has been read to me, and I have had the opportunity to ask questions and all of my questions have been answered to my satisfaction. I consent voluntarily to participate in this research

Name of the main investigator ....
ID No. ....
Tel: ....
Signature: ....
Date: ....

Participant name: ....
ID No.: ....
Tel: ....
Address: ....
Signature: ....
Date: / /
